# Asymmetric and symmetric dimethylarginines and mortality in patients with hematological malignancies—A prospective study

**DOI:** 10.1371/journal.pone.0197148

**Published:** 2018-05-22

**Authors:** Angelika Chachaj, Jerzy Wiśniewski, Justyna Rybka, Aleksandra Butrym, Monika Biedroń, Małgorzata Krzystek-Korpacka, Mariusz Grzegorz Fleszar, Maciej Karczewski, Tomasz Wróbel, Grzegorz Mazur, Andrzej Gamian, Andrzej Szuba

**Affiliations:** 1 Department of Angiology, Wroclaw Medical University, Wroclaw, Poland; 2 Department of Biochemistry, Wroclaw Medical University, Wroclaw, Poland; 3 Department of Haematology, Blood Neoplasms, and Bone Marrow Transplantation, Wroclaw Medical University, Wroclaw, Poland; 4 Department of Physiology, Wroclaw Medical University, Wroclaw, Poland; 5 Department of Internal Medicine, Wroclaw Medical University, Wroclaw, Poland; 6 Department of Mathematics, The Faculty Of Environmental Engineering And Geodesy, Wroclaw University of Environmental and Life Sciences, Wroclaw, Poland; 7 Institute of Immunology and Experimental Therapy, Polish Academy of Sciences, Wroclaw, Poland; University of PECS Medical School, HUNGARY

## Abstract

The study was designed to determine the associations of asymmetric (ADMA) and symmetric (SDMA) dimethylarginines plasma concentrations with all-cause mortality in patients with hematological malignancies. 33 patients with acute myeloid leukemia (AML), 31 patients with non-Hodgkin’s lymphoma (nHL), 32 patients with chronic lymphocytic leukemia (CLL) and 48 patients without malignancy were enrolled into the study. Each patient was followed until death or for at least 14.5 months (range: 14.5–53). Median ADMA and SDMA were significantly elevated in AML, nHL and CLL compared to controls (ADMA: 1.36, 1.24, 1.03, 0.55 μmol/l respectively, p<0.0001; SDMA: 0.86, 0.76, 0.71, 0.52 μmol/l respectively, p<0.0001). High ADMA and SDMA were associated with increased risk for all-cause mortality in CLL group (Hazard ratio (HR) for ADMA: 3.05, 95% CI:1.58–5.88, p = 0.001; HR for SDMA: 4.71, 95% CI:1.91–11.58, p = 0.001). Our study suggests that ADMA and SDMA could be novel prognostic factors for all-cause mortality in CLL patients.

## Introduction

Dimethylarginines, asymmetric (ADMA) and symmetric (SDMA), are the methyl derivates of L-arginine, present in human bloodat micromolar levels. They are the result of degradation of methylated proteins during hydrolytic protein turnover [1].

ADMA inhibits nitric oxide (NO) synthesisvia competitive inhibition of nitric-oxide synthase (NOS) [1,2]. SDMA does not interfere with NOS activity directly but may still have inhibitory effect on NO synthesis by suppressing cellular uptake of its precursor, L-arginine [3,4] and by inhibition of renal tubular absorption of L-arginine [5].

NO has multiple targets in humans, including cardiovascular, nervous and inflammation/immunology systems [6]. NO is an important vasodilator that regulates systemic blood pressure. NO inhibits superoxide generation, the interaction of circulating blood elements with the vessel wall, as well as platelets adhesion and aggregation, smooth muscle cell proliferation and the accumulation of lipids within the vessel wall [7]. Its deficiency results in endothelial dysfunction and atherosclerotic vascular complications. NO may also have a role in the regulation of apoptosis, angiogenesis, cell cycle, invasion and metastasisof malignant cells [8,9].

Increased ADMA plasma levels are linked to endothelial dysfunction, vasoconstriction, elevation of blood pressure and aggravation of experimental atherosclerosis [10]. Elevated plasma ADMA concentrations have been noted in numerous clinical conditions associated with NO-dependent endothelial dysfunction, e.g. hypercholesterolemia [11], hypertension [12], myocardial infarction [13], chronic renal failure [14], chronic heart failure [15], diabetes mellitus [16], homocysteinemia [17] and peripheral arterial disease [18].

SDMA for many years has been considered only as a marker of early renal dysfunction [4]. However, recent studies have demonstrated that both ADMA and SDMA are the markers and mediators of cardiovascular diseases and independent risk factors for all-cause mortality across different populations and methodological approaches [19]. The strongest association between ADMA and all-cause mortality has been observed in critically ill patients from intensive care units (ICU), but the statistically significant relations with all-cause mortality have also been noted in the general population, in patients with renal diseases, peripheral arterial disease and in those with prevalent cardiovascular diseases (CVD) [18,19]. For SDMA, the strongest associations with overall mortality has been demonstrated in the general population [19].

ADMA and SDMA have different clearance pathways.SDMA and partially ADMA are excreted unchanged by the kidney [14] and some amount of ADMA and less of SDMA is a subject of biliary excretion[20,21]. However, the major metabolic pathway of ADMA involves its degradation by dimethylarginine dimethyloaminohydrolase (DDAH) into dimethylamine (DMA) and L-citrulline (CIT) [22–25]. The metabolites of ADMA are excreted in the urine [26]. DDAH has two isoforms: DDAH-1and DDAH-2. DDAH-1 is consider as the main guardian of circulating ADMA and it is strongly expressed in liver and kidney [27–29], but also in pancreas, forebrain, aorta, macrophages and peritoneal neutrophils [30]. DDAH-2 is expressed in vascular endothelium and in immune tissues, including peripheral leukocytes, lymph nodes, spleen and bone marrow[27,30,31].

Reduced DDAH activity and/or expression may contribute to the pathogenesis of NO-dependent endothelial dysfunction in various conditions [15]. High concentrations of glucose [32], homocysteine [33], oxidative stress[22] and its markers, such as oxidized LDL-cholesterol and TNF-α [26], have been shown to suppress DDAH activity. Recent studies also have demonstrated that both ADMA and SDMA can be Nα-acetylated to form asymmetric and symmetric Nα-acetyldimethylarginine (Ac-ADMA and Ac-SDMA) which are than excreted by the kidney [34].

We have reported earlier that plasma ADMA concentrations were significantly elevated in patients with different hematological malignancies [35]. The study of Doroszko et al. demonstrated elevated plasma ADMA concentrations in children with acute lymphoblastic leukemia (ALL)[36].

Plasma ADMA and SDMA concentrations and their prognostic value have not been studied separately in individual hematological malignancies in adults. Also, the pathophysiological mechanism of this elevation remains unclear.

The current study was designed to prospectively determine associations of circulating ADMA and SDMA concentrations with all-cause mortality in patients with three different hematological malignancies: acute myeloid leukemia (AML), non-Hodgkin's lymphomas (nHL) and chronic lymphocytic leukemia (CLL).

We have also determined concentrations of ADMA metabolites (DMA and CIT) to estimate DDAH activity. Endothelial function was assessed measuring serum concentration of soluble vascular cell adhesion molecule-1 (sVCAM-1), an established marker of endothelial dysfunction in a number of pathological conditions [37].

## Materials and methods

### Patients and controls

The study group included 98 consecutive patients of the Hematology Clinic of Wroclaw Medical University in Poland with three different hematological malignancies: 33 patients with acute myeloid leukemia (AML), 31 patients with non Hodgkin’s lymphoma (nHL) and 32 patients with chronic lymphocytic leukemia (CLL). They were recruited from February 2011 to May 2014. All recruited patients were in the active phase of the disease prior to the initiation of chemotherapy and without prior history of any neoplastic disease. All the plasma and serum samples were taken before chemotherapy was started.

Complete clinical and follow-up data were obtained for all the patients. Clinical evaluation of all patients included age, sex, height and weight, co-morbidities (arterial hypertension, diabetes, hypercholesterolemia and obesity), smoking history and basic laboratory test results.

Body mass index (BMI) was calculated as weight (kg) divided by the square of height (m^2^). Survival time in hematological groups was estimated in months and calculated from the date of venous blood sampling to the date of death. The mean time from blood collection to the end of the follow-up was 33.86 months (range: 14.5–53 months).

The control group consisted of 48 subjects without malignancy recruited from the University Family Practice and the Department of Internal Medicine of Wroclaw Medical University in Poland. They were matched for age, sex, co-morbidities (arterial hypertension, diabetes, hypercholesterolemia, obesity) and smoking habits with the hematological groups.

The study was approved by the Bioethical Committee of Wroclaw Medical University (approval No. KB-41/2011) and adhered to the principles of the Declaration of Helsinki. The informed written consent was obtained from each study subject.

### Biochemical analysis

Venous peripheral blood samples were collected after an overnight fast using the Sarstedt S-Monovette system (for plasma: S-Monovette 4.9 ml EDTA, for serum: S-Monovette 4.9 ml,Sarstedt AG & Co., Nümbrecht, Germany). Blood samples were centrifuged (1000 x g for 15 minutes at 4°C).

Plasma samples for arginine derivates and ADMA metabolites and serum samples for sVCAM-1 from all the study subjects were initially frozen at -20°C (up to 2–3 weeks) and then were stored at -80°C until the analysis.

Basic laboratory tests were carried out only in hematological groups andwere performed immediately after venous blood collection.They included: complete blood count(white blood cells (WBC),red blood cells (RBC), hemoglobinplatelets (PLT)), C-reactive protein (CRP), tumor burden (activity of lactate dehydrogenase (LDH), uric acid), renal function (serum creatinine level, urea), hepatic function tests (aspartate transaminase (AST) and alanine transaminase (ALT) activity, total serum bilirubin level, gamma-glutamyltransferase activity (GGT), alkaline phosphatase, total protein, albumin), coagulation/fibrinolysis function (APTT, INR, fibrinogen, D-dimer), lipid profile (total cholesterol, triglycerides) and β2-microglobulin level. These laboratory parameters were determined by XN 2000Analyser from Sysmex (complete blood count), Architect ci4000 Clinical Chemistry Analyzer from Abbott Laboratories (biochemical studies) and ACL TOP 300 from Werfen (haemostasis parameters).

Glomerular filtration rate (GFR) was calculated from the simplified equation developed using the Modification of Diet in Renal Disease (MDRD) formula [38].

Plasma concentrations of ADMA, SDMA and L-arginine were measured by high-performance liquid chromatography (HPLC) after precolumn derivatization with o-phthaldialdehyde (OPA), as previously described [11].

Plasma concentrations of two metabolites of ADMA: CIT and DMA were measured using liquid chromatography combined with mass spectrometry method (LC-MS/MS). L-arginine, ADMA, SDMA, DMA and CIT levels were expressed in μmol/L.

Serum concentrations of sVCAM-1were measured using ELISA method according to the manufacturer’s guidelines (Diaclone, France) and expressed in μg/mL.

### Statistical analysis

Baseline demographic and medical characteristics with normal distribution were reported as mean and standard deviation [±SD] and categorical variables were reported as number and percentage. Biochemical laboratory values were presented as mean and [±SD].

Statistical analysis of age and BMI were calculated using Kruskall-Wallis test. Differences among study groups in sex and co-morbidities were estimated using Fisher's Exact Test.

Not normally distributed characteristics were presented as median with the interquartile range (Q1–Q3). The non-parametric Mann–Whitney test was used for statistical analysis of variables which were not normally distributed: L-arginine, ADMA, SDMA, ADMA metabolites and sVCAM-1. Post-hoc analysis was performed using multiple comparisons method with the Holm’s correction.

The influence of the concentrations of ADMA and SDMA at the risk of death was calculated using Cox regression analysis. The Kaplan–Meier method was used to appoint a survival curves. Values of ADMA/SDMA higher than mean value,estimated separately in each hematological group, were considered “high” in this method.

An univariate and multivariate analysis using Cox regression was performed to determine the independent effect of ADMA and SDMA on mortality of CLL patients. Given small statistical power caused by small sample size of each group instead of separate small models we decided to use a model for all hematological patients with group as a factor (with CLL diagnosis as a baseline), and with interaction term between ADMA/SDMA and group. Separate models were created for ADMA and SDMA. Other variables included in the models were as follows: age, WBC, GFR, diagnosis of hypertension, diabetes, and the group. β-2 microglobulin and LDH were not taken into account as elements of these multivariate models because of the amount of the missing data.

The correlations between ADMA, SDMA, L-arginine, DMA, CIT and sVCAM-1, and between these parameters and the results of basic laboratory tests were calculated using Spearman’s rank correlation test.

In all the calculations p ≤ 0.05 was considered as statistically significant. The statistical analysis was performed using the R package for Windows (version 3.3.2).

## Results

There were no significant differences between the study groups in relation to age, sex and BMI (p>0.05). Baseline demographic and medical characteristics of all the hematological groups and controls are presented in Table 1.

**Table 1:** Selected demographic and medical parameters in the study groups.

parameter	AML (n = 33)	nHL (n = 31)	CLL (n = 32)	controls (n = 48)	statistics
**age (years) mean, range [±SD]**	65.5 (25–86) [±13.1]	62.2 (28–83) [±14.2]	62.8 (23–90) [±12.4]	62.5 (38–92) [±14]	chi^2^ = 2.11 p = 0.55
**male gender [n (%)]**	15 (45%)	14 (47%)	16 (52%)	22 (46%)	p = 0.964
**body mass index (BMI) [±SD] [kg/m**^**2**^**]**	27.3 [±4.6]	27.0 [±6.2]	26.2 [±7.0]	28.9 [±4.9]	chi^2^ = 6.38 p = 0.095
**obesity (BMI>30kg/m**^**2**^**) [n (%)]**	7 (21%)	5 (17%)	3 (10%)	18 (38%)	p = 0.792
**hypertension [n (%)]**	14 (42%)	10 (33%)	10 (32%)	27 (56%)	p = 0.316
**diabetes [n (%)]**	7 (21%)	5 (17%)	1 (3%)	10 (21%)	p = 0.261
**hypercholesterolemia [n (%)]**	0	1 (3%)	3 (10%)	13 (27%)	p = 0.0022
**cigarette smokers [n (%)]**	8 (24%)	4 (13%)	3 (10%)	5 (10%)	p = 0.172

Basic biochemical laboratory values in the hematological groups are presented in Table 2.

**Table 2:** Basic clinical laboratory values in the hematological groups.

parameter	AML (n = 33) mean values [±SD]	nHL (n = 31) mean values [±SD]	CLL (n = 32) mean values [±SD]
**WBC [10**^**3**^**/μl]**	46.6 [±85.9]	14.7 [±17.0]	83.3 [±173.8]
**RBC [10**^**6**^**/μl]**	3.0 [±0.4]	4.3 [±0.9]	4.3 [±0.7]
**hemoglobin [g/dL]**	9.3 [±1.2]	11.9 [±2.8]	13.1 [±1.8]
**PLT [10**^**3**^**/μl]**	83.1 [±91.6]	257.4 [±124.7]	188.1 [±69.8]
**CRP [mg/l]**	59.2 [±55.6]	40.5 [±61.8]	12.8 [±28.9]
**LDH [U/l]**	615.5 [±717.1]	421.8 [±467.3]	316.7 [±335.0]
**creatinine [mg/dl]**	0.9 [±0.3]	1.0 [±0.7]	0.9 [±0.3]
**GFR [ml/min/1,73m**^**2**^**]**	83.5 [±29.5]	88.1 [±46.6]	85.8 [±23.8]
**urea [mg/dl]**	36.3 [±12.6]	33.9 [±9.5]	33.9 [±12.2]
**uric acid [mg/dl]**	5.5 [±1.8]	6.1 [±2.1]	6.1 [±1.6]
**fibrinogen [g/l]**	4.1 [±1.2]	3.6 [±1.5]	3.1 [±1.2]
**INR**	1.3 [±0.3]	1.0 [±0.1]	1.0 [±0.1]
**D-dimers [μg/ml]**	3.9 [±6.7]	1.9 [±1.5]	0.8 [±1.5]
**APTT [sek.]**	33.2 [±10.7]	29.9 [±6.0]	26.8 [±9.0]
**AST [U/l]**	35.5 [±28.9]	29.7 [±17.3]	25.8 [±13.7]
**ALT [U/l]**	42.5 [±36.7]	23.6 [±16.4]	23.4 [±10.7]
**total bilirubin [mg/dl]**	0.9 [±1.0]	0.8 [±0.7]	0.6 [±0.3]
**GGT [U/l]**	115.7 [±148.8]	29.3 [±9.3]	45.4 [±41.1]
**total cholesterol [mg/dl]**	127.3 [±45.4]	180.4 [±32.1]	185.5 [±63.0]
**triglycerides [mg/dl]**	144.7 [±98.7]	179.8 [±66.5]	84.7 [±28.7]
**total protein [g/dl]**	6.8 [±0.8]	6.9 [±0.9]	6.7 [±1.7]
**albumin [g/dl]**	3.9 [±0.4]	3.8 [±0.8]	4,3 [±0,3]
**B2-microglobulin [mg/l]**	-	2.9 [±1.7]	4.6 [±6.7]
**alkaline phosphatase [U/l]**	168.5 [±141.7]	120.7 [±69.7]	104.9 [±63.6]

WBC—white blood cells; RBC—red blood cells; PLT—plates; CRP- C-reactive protein; LDH—lactate dehydrogenase; GFR—glomerular filtration rate; AST—aspartate transaminase; ALT—alanine transaminase; GGT—gamma-glutamyltransferase.

### The influence of ADMA on the risk of all-cause mortality

Univariate Cox regression analysis and Kaplan-Meier analysis demonstrated that higher ADMA and SDMA levels were associated with increased risk for all-cause mortality in CLL group (hazard ratio (HR) for ADMA: 3.05, 95% confidence interval: 1.58–5.88, p = 0.001, HR for SDMA: 4.71, 95% confidence interval:1.91–11.58, p = 0.001). The subgroups of low-ADMA, as well as of low-SDMA within CLL group had no recorded deaths.

In AML and nHL groups univariate Cox regression analysis and Kaplan-Meier analysis did not reveal statistically significant associations between ADMA or SDMA levels and all-cause mortality rate.

**Fig 1:**
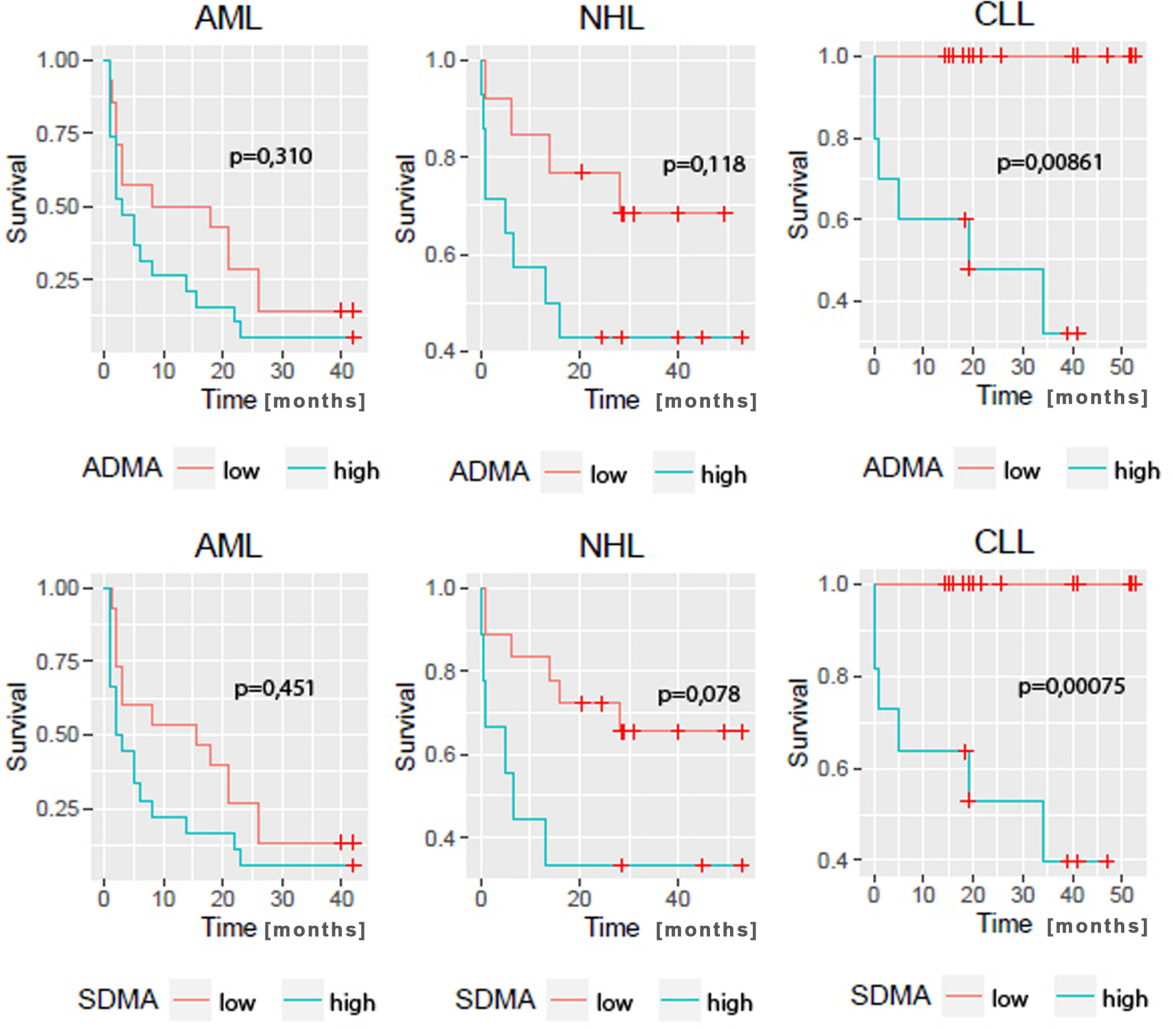
Kaplan–Meier survival curve analysis in relation to ADMA and SDMA in hematological groups. p was calculated using Cox regression analysis.

Fig 1 presents Kaplan–Meier survival curve analysis in relation to ADMA and SDMA in hematological groups.

Multivariate Cox models, separately for ADMA and SDMA,showed significant main effect of ADMA, SDMA, diabetes and difference between CLL and AML group. In addition, SDMA model showed significant influence of WBC. Interaction effect in SDMA model showed significantly stronger effect of SDMA on mortality in CLL group when compared to AML group. Interaction term in ADMA model was not statistically significant, however, its positive effect on the model fit was a decisive factor to keep it.

The results of multivariate analysis are presented in Tables 3 and 4.

**Table 3:** Multivariate analysis with ADMA and with CLL diagnosis as a baseline.

parameter	coefficient	standard error	p-value
**ADMA**	1.16	0.54	0.031*
**Age**	0.02	0.02	0.31
**WBC**	<0.01	<0.01	0.98
**GFR**	<0.01	<0.01	0.51
**hypertension**	-0.28	0.36	0.44
**diabetes**	1.25	0.43	0.004*
**AML group**	3.26	1.11	0.003*
**nHL group**	1.44	1.30	0.27
**ADMA : AML group interaction**	-0.94	0.50	0.059
**ADMA : nHL group interaction**	-0.54	0.65	0.41

*—statistically relevant

**Table 4:** Multivariate analysis with SDMA and with CLL diagnosis as a baseline.

	coefficient	standard error	p-value
**SDMA**	1.80	0.51	<0.001*
**age**	0.02	0.02	0.22
**WBC**	<0.01	<0.01	0.008*
**GFR**	<0.01	<0.01	0.18
**hypertension**	-0.25	0.36	0.47
**diabetes**	1.31	0.44	0.003*
**AML group**	3.97	1.18	<0.001*
**nHL group**	1.98	1.21	0.10
**SDMA : AML group interaction**	-1.66	0.62	0.007*
**SDMA : nHL group interaction**	-0.92	0.62	0.139

*—statistically relevant

### Comparisons of arginine derivates, ADMA metabolites and sVCAM-1

Plasma ADMA levels did not correlate with age and was not associated with sex, either in the group of all the study subjects, or in the study groups separately.

Median and range quartile (Q1—Q3) plasma levels of ADMA, SDMA, L-arginine, DMA, CIT and sVCAM-1 in hematological groups and in control group are summarized in Table 5.

**Table 5:** Median and range quartile (Q1—Q3) plasma levels of ADMA, SDMA, L-arginine, DMA, CIT and serum levels of sVCAM-1 in the studied groups.

parameter	AML (n = 33) median value (Q1—Q3)	nHL (n = 31) median value (Q1—Q3)	CLL (n = 32) median value (Q1—Q3)	controls (n = 48) median value (Q1—Q3)	p value
**ADMA (μmol/l)**	1.36 (1.03–1.88)*	1.24 (0.83–1.56)^	1.03 (0.92–1.40)^#^	0.55 (0.49–0.60)*^^#^	<0.0001
**SDMA (μmol/l)**	0.86 (0.69–1.16)*	0.76 (0.54–1.33)^	0.71 (0.57–0.95)^#^	0.52 (0.43–0.58)*^^#^	<0.0001
**L-arginine (μmol/l)**	52.42 (40.73–57.90)*	54.14 (44.95–70.24)	66.81 (55.97–6.20)*^	55.69 (47.63–61.14)^	0.005
**L-arginine/ADMA**	29.08 (21.68–45.69)*^&^	50.14 (32.58–65.26)^	64.69 (41.94–75.53)^#&^	101.02 (86.95–113.94)*^^#^	<0.0001
**DMA (μmol/l)**	8.27 (5.82–9.92)*	10.58 (8.80–13.53)*^	10.51 (6.65–12.04)	9.11 (7.52–10.01)^	0.001
**CIT (μmol/l)**	22.01 (15.03–28.31)* ^#&^	29.94 (23.18–35.68)^^#+^	36.13 (31.23–40.08)^&+^	36.09 (30.14–46.20)*^	<0.0001
**sVCAM-1 (μg/ml)**	1.64 (1.15–2.02)*	2.08 (1.16–3.02)^	1.26 (1.06–2.09)^#^	0.94 (0.76–1.12)*^^#^	<0.0001

*^#&+—statistically significant differences between the groups (post-hoc analysis)

Median ADMA and SDMA plasma concentrations were significantly higher in hematological groups than in control group.

Median L-arginine plasma level was significantly higher in CLL group than in AML and control groups. Control group has significantly higher ratio of L-arginine to ADMA in comparison to the other groups. In addition, CLL group has a significantly higher ratio of L-arginine to ADMA than AML.

Median DMA plasma level was significantly higher in nHL group in comparison to AML and control groups.

Median CIT plasma level was significantly lower in AML group in comparison to all other groups. Additionally, median CIT plasma level in NHL group was significantly lower than in CCL and control groups.

Median sVCAM-1serum level in control group was significantly lower in comparison to all hematological groups.

### Correlations betweenADMA, SDMA, L-arginine, ADMA metabolites and sVCAM-1 in the study groups

Correlations between ADMA/SDMA and L-arginine, SDMA, ADMA metabolites and sVCAM-1 in the study groups are presented in Tables 6 and 7.

**Table 6:** Correlations between ADMA and L-arginine, SDMA, ADMA metabolites and sVCAM-1 in the study groups (Spearman’s rank correlation test).

	L-arginine	SDMA	DMA	CIT	sVCAM-1
**ADMA in AML group**	ns	r = 0.74 p<0.0001	ns	ns	r = 0.49 p = 0.004
**ADMA in nHL group**	ns	r = 0.72 p<0.0001	r = 0.39 p = 0.031	ns	r = 0.59 p = 0.001
**ADMA in CLL group**	ns	r = 0.57 p = 0.001	ns	r = -0.39 p = 0.043	r = 0.45 p = 0,031
**ADMA in control group**	ns	ns	ns	ns	ns

ns- statistically not relevant

**Table 7:** Correlations between SDMA and L-arginine, ADMA, its metabolites and sVCAM-1 in the study groups (Spearman’s rank correlation test).

	L-arginine	ADMA	DMA	CIT	sVCAM-1
**SDMA in AML group**	ns	r = 0.74 p<0.0001	r = 0.63 p = 0.0001	ns	r = 0.49 p = 0.004
**SDMA in nHL group**	ns	r = 0.72 p<0.0001	r = 0.55 p = 0.002	ns	r = 0.54 p = 0.002
**SDMA in CLL group**	ns	r = 0.57 p = 0.001	ns	r = - 0.63 p = 0.001	ns
**SDMA in control group**	r = - 0.34 p = 0.019	ns	ns	ns	r = 0.33 p = 0.022

ns- statistically not relevant

Correlations between DMA and CIT and between DMA/CIT and L-arginine and sVCAM-1 are presented in S1 Table.

#### Correlations between ADMA and SDMA and biochemical parameters in the study groups

Statistically relevant correlations between ADMA and SDMA and the results of standard laboratory tests are presented in Table 8.

**Table 8:** Statistically relevant correlations between ADMA and SDMA and the results of standard laboratory tests (Spearman’s rank correlation test).

group	statistically relevant correlations		statistics	
			r	p
AML	ADMA and	WBC	0.79	<0.0001
		LDH	0.79	<0.0001
		AST	0.45	0.010
		triglycerides	0.65	0.016
nHL		urea	0.52	0.048
		uric acid	0.48	0.008
		AST	0.37	0.046
		RBC	-0.38	0.041
		hemoglobin	-0.52	0.003
		fibrinogen	-0.40	0.046
CLL		WBC	0.46	0.012
		LDH	0.46	0.019
		D-dimer	0.59	0.011
		GGT	0.63	0.017
		β-2 microglobulin	0.61	0.026
		RBC	-0.43	0.022
		hemoglobin	-0.40	0.030
		APTT	-0.49	0.037
AML	SDMA and	WBC	0.66	<0.0001
		LDH	0.56	0.005
		uremic acid	0.50	0.004
		AST	0.38	0.033
		GGT	0.58	0.024
		triglycerides	0.60	0.029
		albumin	-0.58	0.015
		total cholesterol	-0.54	0.016
nHL		CRP	0.41	0.027
		creatinine	0.40	0.034
		uric acid	0.59	0.001
		hemoglobin	-0.39	0.031
		albumin	-0.67	0.003
CLL		LDH	0.61	0.001
		creatinine	0.61	0.002
		uric acid	0.44	0.040
		D-dimer	0.50	0.035
		alkaline phosphatase	0.60	0.014
		RBC	-0.43	0.022
		hemoglobin	-0.49	0.006
		GFR	-0.65	0.0001

WBC—white blood cells; LDH—lactate dehydrogenase; AST—aspartate transaminase; RBC—red blood cells; GGT—gamma-glutamyltransferase; CRP- C-reactive protein; GFR—glomerular filtration rate.

## Discussion

The relevant findings of the present study were as follows. (1) Plasma concentrations of ADMA and SDMA in AML, nHL and CLL were significantly higher than in controls. (2) Elevated ADMA and SDMA were associated with increased risk for all-cause mortality in CLL group. (3) Pathophysiology of ADMA and SDMA elevation in hematological malignancies may have complex origin. It is probably an increased production rate due to extensive protein catabolism and possibly decreased metabolisms as a result of impaired DDAH activity. (4) Associations between ADMA and SDMA concentrations and laboratory basic parameters were different among hematological groups.

ADMA concentrations were generally at least twofold higher in hematological groups compared to the control group. ADMA levels in hematological groups in comparison to the reference range of ADMA assessed in healthy adults and determined by a recent meta-analysis [39] are also significantly higher. Such high ADMA concentrations were reported only in patients with end stage renal disease [40,41] and in critically ill ICUpatients [42]. In cardiovascular patients reported ADMA levels are smaller[43,44].

Among hematological malignancies, the highest level of ADMA was observed in patients with AML, median—in the group with nHL and the lowest—in CLL group. Differences in SDMA concentrations between the study groups were similar to ADMA concentrations, although the differences between the groups were less pronounced than ADMA. We presume that different clearance pathways of ADMA and SDMA may result in smaller increase of SDMA than ADMA in hematological malignancies. SDMA is mainly excreted by the kidneys in unchanged form. In our patients renal function was not significantly affected. In contrast, ADMA is mainly metabolized by DDAH and the activity of this enzymemay affect ADMA concentrations.

The study of Doroszko et al. demonstrated that elevated plasma ADMA concentrations in children with acute lymphoblastic leukemia (ALL) prior to the treatment were associated with endothelial dysfunction assessed by a laser Doppler flowmeter and that ALL treatment led to reduction of ADMA levels and recovery of endothelial function [36].

L-arginine/ADMA ratio is suggested to be important determinant of NOS activity, thus, indirectly, of NO bioavailability [45]. NO deficiency is one of the first indicator of endothelial dysfunction and precedes vascular complications [6]. In our study, L-arginine/ADMA ratio in hematological groups were significantly lower in comparison to the control group. The lowest L-arginine/ADMA ratio was in AML group.

sVCAM-1 significantly elevated in hematological groups in our study also suggests endothelial dysfunction in hematological cancers. Positive correlations between ADMA and sVCAM-1 levels confirms close relationship between ADMA, sVCAM-1 and endothelial dysfunction. sVCAM-1 is recognized as a marker of endothelial dysfunction in many conditions [37], and we have recently confirmed correlation between endothelial dysfunction and sVCAM-1 in children with ALL (unpublished data). Malignancy associated inflammation may cause increase in sVCAM-1 serum concentrations, however in our study no correlation between sVCAM-1 and CRP in any hematological group was found. Similarly, there were no correlations between ADMA and CRP in any hematological group.

Endothelial dysfunction and its significance in the pathogenesis of hematological malignancies certainly requires further elucidation.

Our study demonstrates for the first time that high ADMA and SDMA plasma concentrations were the predictors of increased risk for all-cause mortality in CLL group. The subgroups of low-ADMA and of low-SDMA within CLL group had no recorded deaths. Multivariate analysis Cox models separately for ADMA and SDMA showed significant main effect of ADMA, SDMA, diabetes, GFR and difference between CLL and AML group. In addition, SDMA model showed significant influence of WBC.

Differences in all-cause mortality rate in respect to ADMA and SDMA concentrations in the groups of AML and nHL were not statistically significant. Although, in nHL group the significance of the association between SDMA and survival was close to the statistical threshold. Nevertheless, the role of ADMA and SDMA in hematological cancers deserves further studies.

Increased plasma levels of ADMA and SDMA in the hematological malignancies may have complex origin. Primarily, they are probably the result of increased production due to extensive protein catabolism that is characteristic for hematological cancers. ADMA and SDMA originate from the hydrolysis of nuclear proteins containing post-translationally methylated arginine residues [1]. The significant correlations between ADMA and SDMA and WBC and LDH in AML and CLL groups and between ADMA and SDMA and uric acid in nHL group might indicate such association. All hematological patients were in active phase of the disease and prior to initiation of chemotherapy.

ADMA elevation could also result from downregulation or inhibition of DDAH, the enzymethat degrades ADMA to DMA and CIT. We hypothesize that DDAH activity may be inhibited by oxidative stress [26]. Malignant cells often demonstrate an abnormal redox metabolism associated with down-regulation of antioxidant enzymes and mitochondrial dysfunction [46]. ADMA elevation may further exacerbate oxidative stress by eNOS uncoupling and thus contributing to endothelial dysfunction [47,48]. Relatively low values of DMA and CIT in our hematological groups in relation to ADMA concentrations, especially in the AML group, and greater levels of ADMA than SDMA might suggest DDAH inhibition. However, DMA and CIT can be derived from many different pathways and thus cannot be considered reliable markers of ADMA-metabolism. Further studies are needed to determine relevance of our hypothesis regarding inhibition of DDAH activity in hematological malignancies.

Other factors that could contribute to elevation of ADMA and SDMA are impaired liver and renal function. Although in studied hematological patients liverand renal functions were not significantly affected (Table 2), ADMA concentrations correlated positively with AST (in AML and nHL) and with GGT (in CLL) and SDMA concentrations in AML correlated with AST and GGT. That might indicate the significance of the liver function in ADMA and SDMA elimination and is in agreement with recent studies [21]. Further, associations between SDMA and creatinine (in nHL and CLL groups), between DMA or CIT and creatinine (in AML and nHL groups)and probably associations between SDMA and DMA (in AML and nHL groups) or SDMA and CIT (in CLL group) suggeststhat concentrationsof SDMA, DMA and CIT depend on renal function.

In conclusion, to our knowledge this is the first study demonstrating that ADMA and SDMA may serve as a novel prognostic factorsfor all-cause mortality in patients with CLL. However, our study was conducted on relatively small number of patients. While the data are highly suggestive, an independent and causal association of ADMA/SDMA and outcome in CLL remains to be shown in larger studiesto validate our findings and to determine the exact role of ADMA and SDMA elevation in hematological cancers.

## Supporting information

S1 Table Correlations between DMA and CIT and between DMA/CIT and L-arginine and sVCAM-1 in the study groups (Spearman’s rank correlation test). ns- statistically not relevant
(DOCX)

S1 Data Raw data. This spreadsheet includes all the study individual parameters.
(XLSX)

## References

[1] Boger RH. Asymmetric dimethylarginine: understanding the physiology, genetics, and clinical relevance of this novel biomarker. Proceedings of the 4th International Symposium on ADMA. Pharmacol Res. 2009;60:447. 10.1016/j.phrs.2009.10.001 19853807

[2] Boger RH, Vallance P, Cooke JP. Asymmetric dimethylarginine (ADMA): a key regulator of nitric oxide synthase. Atheroscler Suppl. 2003 12;4(4):1–3. 14664896 10.1016/s1567-5688(03)00027-8

[3] Closs EI, Basha FZ, Habermeier A, Forstermann U. Interference of L-arginine analogues with L-arginine transport mediated by the y+ carrier hCAT-2B. Nitric Oxide. 1997 2;1(1):65–73. doi: 10.1006/niox.1996.0106 9701046 10.1006/niox.1996.0106

[4] Schwedhelm E, Boger RH. The role of asymmetric and symmetric dimethylarginines in renal disease. Nat Rev Nephrol. 2011 5;7(5):275–85. doi: 10.1038/nrneph.2011.31 21445101 10.1038/nrneph.2011.31

[5] Tojo A, Welch WJ, Bremer V, Kimoto M, Kimura K, Omata M, et al Colocalization of demethylating enzymes and NOS and functional effects of methylarginines in rat kidney. Kidney Int. 1997 12;52(6):1593–601. 9407505 10.1038/ki.1997.490

[6] Moncada S, Higgs EA. The discovery of nitric oxide and its role in vascular biology. Br J Pharmacol. 2006 1;147 Suppl 1:S193–201. 16402104 10.1038/sj.bjp.0706458 PMC1760731

[7] Forstermann U, Sessa WC. Nitric oxide synthases: regulation and function. Eur Heart J. 2012 4;33(7):829–37, 37a-37d. doi: 10.1093/eurheartj/ehr304 21890489 10.1093/eurheartj/ehr304 PMC3345541

[8] Choudhari SK, Chaudhary M, Bagde S, Gadbail AR, Joshi V. Nitric oxide and cancer: a review. World J Surg Oncol. 2013 5 30;11:118 doi: 10.1186/1477-7819-11-118 23718886 10.1186/1477-7819-11-118 PMC3669621

[9] Scicinski J, Oronsky B, Ning S, Knox S, Peehl D, Kim MM, et al NO to cancer: The complex and multifaceted role of nitric oxide and the epigenetic nitric oxide donor, RRx-001. Redox Biol. 2015 12;6:1–8. doi: 10.1016/j.redox.2015.07.002 26164533 10.1016/j.redox.2015.07.002 PMC4529402

[10] Boger RH, Cooke JP, Vallance P. ADMA: an emerging cardiovascular risk factor. Vasc Med. 2005 7;10 Suppl 1:S1–2. 10.1177/1358836X0501000101 16444862

[11] Boger RH, Bode-Boger SM, Szuba A, Tsao PS, Chan JR, Tangphao O, et al Asymmetric dimethylarginine (ADMA): a novel risk factor for endothelial dysfunction: its role in hypercholesterolemia. Circulation. 1998 11 3;98(18):1842–7. 9799202 10.1161/01.cir.98.18.1842

[12] Perticone F, Sciacqua A, Maio R, Perticone M, Galiano Leone G, Bruni R, et al Endothelial dysfunction, ADMA and insulin resistance in essential hypertension. Int J Cardiol. 2010 7 23;142(3):236–41. doi: 10.1016/j.ijcard.2008.12.131 19168237 10.1016/j.ijcard.2008.12.131

[13] Sen N, Ozlu MF, Akgul EO, Kanat S, Cayci T, Turak O, et al Elevated plasma asymmetric dimethylarginine level in acute myocardial infarction patients as a predictor of poor prognosis and angiographic impaired reperfusion. Atherosclerosis. 2011 11;219(1):304–10. doi: 10.1016/j.atherosclerosis.2011.06.021 21726864 10.1016/j.atherosclerosis.2011.06.021

[14] Boger RH, Zoccali C. ADMA: a novel risk factor that explains excess cardiovascular event rate in patients with end-stage renal disease. Atheroscler Suppl. 2003 12;4(4):23–8. 14664899 10.1016/s1567-5688(03)00030-8

[15] Boger RH. Asymmetric dimethylarginine, an endogenous inhibitor of nitric oxide synthase, explains the "L-arginine paradox" and acts as a novel cardiovascular risk factor. J Nutr. 2004 10;134(10 Suppl):2842S–7S; discussion 53S. doi: 10.1093/jn/134.10.2842S 15465797 10.1093/jn/134.10.2842S

[16] Abbasi F, Asagmi T, Cooke JP, Lamendola C, McLaughlin T, Reaven GM, et al Plasma concentrations of asymmetric dimethylarginine are increased in patients with type 2 diabetes mellitus. Am J Cardiol. 2001 11 15;88(10):1201–3. 11703973 10.1016/s0002-9149(01)02063-x

[17] Dayal S, Lentz SR. ADMA and hyperhomocysteinemia. Vasc Med. 2005 7;10 Suppl 1:S27–33. 16444866 10.1191/1358863x05vm599oa

[18] Boger RH, Endres HG, Schwedhelm E, Darius H, Atzler D, Luneburg N, et al Asymmetric dimethylarginine as an independent risk marker for mortality in ambulatory patients with peripheral arterial disease. J Intern Med. 2011 3;269(3):349–61. doi: 10.1111/j.1365-2796.2010.02322.x 21175900 10.1111/j.1365-2796.2010.02322.x

[19] Schlesinger S, Sonntag SR, Lieb W, Maas R. Asymmetric and Symmetric Dimethylarginine as Risk Markers for Total Mortality and Cardiovascular Outcomes: A Systematic Review and Meta-Analysis of Prospective Studies. PLoS One. 2016;11(11):e0165811 doi: 10.1371/journal.pone.0165811 27812151 10.1371/journal.pone.0165811 PMC5094762

[20] Ferrigno A, Di Pasqua LG, Berardo C, Rizzo V, Richelmi P, Vairetti M. Changes in Biliary Levels of Arginine and its Methylated Derivatives after Hepatic Ischaemia/Reperfusion. Basic Clin Pharmacol Toxicol. 2016;119:101–9. doi: 10.1111/bcpt.12540 26663642 10.1111/bcpt.12540

[21] Ferrigno A, Di Pasqua LG, Berardo C, Richelmi P, Vairetti M. Liver plays a central role in asymmetric dimethylarginine-mediated organ injury. World J Gastroenterol. 2015;21:5131–7. doi: 10.3748/wjg.v21.i17.5131 25954086 10.3748/wjg.v21.i17.5131 PMC4419053

[22] Murray-Rust J, Leiper J, McAlister M, Phelan J, Tilley S, Santa Maria J, et al Structural insights into the hydrolysis of cellular nitric oxide synthase inhibitors by dimethylarginine dimethylaminohydrolase. Nat Struct Biol. 2001;8:679–83. doi: 10.1038/90387 11473257 10.1038/90387

[23] Chobanyan K, Mitschke A, Gutzki FM, Stichtenoth DO, Tsikas D. Accurate quantification of dimethylamine (DMA) in human plasma and serum by GC-MS and GC-tandem MS as pentafluorobenzamide derivative in the positive-ion chemical ionization mode. J Chromatogr B Analyt Technol Biomed Life Sci. 2007;851:240–9. doi: 10.1016/j.jchromb.2007.03.006 17400039 10.1016/j.jchromb.2007.03.006

[24] Ghebremariam YT, Erlanson DA, Yamada K, Cooke JP. Development of a dimethylarginine dimethylaminohydrolase (DDAH) assay for high-throughput chemical screening. J Biomol Screen. 2012;17:651–61. doi: 10.1177/1087057112441521 22460174 10.1177/1087057112441521 PMC3606823

[25] Tsikas D, Thum T, Becker T, Pham VV, Chobanyan K, Mitschke A, et al Accurate quantification of dimethylamine (DMA) in human urine by gas chromatography-mass spectrometry as pentafluorobenzamide derivative: evaluation of the relationship between DMA and its precursor asymmetric dimethylarginine (ADMA) in health and disease. J Chromatogr B Analyt Technol Biomed Life Sci. 2007;851:229–39. doi: 10.1016/j.jchromb.2006.09.015 17011246 10.1016/j.jchromb.2006.09.015

[26] Ito A, Tsao PS, Adimoolam S, Kimoto M, Ogawa T, Cooke JP. Novel mechanism for endothelial dysfunction: dysregulation of dimethylarginine dimethylaminohydrolase. Circulation. 1999;99:3092–5. 10377069 10.1161/01.cir.99.24.3092

[27] Palm F, Onozato ML, Luo Z, Wilcox CS. Dimethylarginine dimethylaminohydrolase (DDAH): expression, regulation, and function in the cardiovascular and renal systems. Am J Physiol Heart Circ Physiol. 2007;293:H3227–45. doi: 10.1152/ajpheart.00998.2007 17933965 10.1152/ajpheart.00998.2007

[28] Nijveldt RJ, Teerlink T, Siroen MP, van Lambalgen AA, Rauwerda JA, van Leeuwen PA. The liver is an important organ in the metabolism of asymmetrical dimethylarginine (ADMA). Clin Nutr. 2003;22:17–22. 12553945 10.1054/clnu.2002.0612

[29] Nijveldt RJ, Teerlink T, van Guldener C, Prins HA, van Lambalgen AA, Stehouwer CD, et al Handling of asymmetrical dimethylarginine and symmetrical dimethylarginine by the rat kidney under basal conditions and during endotoxaemia. Nephrol Dial Transplant. 2003;18:2542–50. 14605276 10.1093/ndt/gfg452

[30] Tran CT, Fox MF, Vallance P, Leiper JM. Chromosomal localization, gene structure, and expression pattern of DDAH1: comparison with DDAH2 and implications for evolutionary origins. Genomics. 2000;68:101–5. doi: 10.1006/geno.2000.6262 10950934 10.1006/geno.2000.6262

[31] Kimoto M, Whitley GS, Tsuji H, Ogawa T. Detection of NG,NG-dimethylarginine dimethylaminohydrolase in human tissues using a monoclonal antibody. J Biochem. 1995;117:237–8. 7608105 10.1093/jb/117.2.237

[32] Sorrenti V, Mazza F, Campisi A, Vanella L, Li Volti G, Di Giacomo C. High glucose-mediated imbalance of nitric oxide synthase and dimethylarginine dimethylaminohydrolase expression in endothelial cells. Curr Neurovasc Res. 2006;3:49–54. 16472125 10.2174/156720206775541778

[33] Stuhlinger MC, Tsao PS, Her JH, Kimoto M, Balint RF, Cooke JP. Homocysteine impairs the nitric oxide synthase pathway: role of asymmetric dimethylarginine. Circulation. 2001;104:2569–75. 11714652 10.1161/hc4601.098514

[34] Rodionov RN, Martens-Lobenhoffer J, Brilloff S, Burdin DV, Jarzebska N, Demyanov AV, et al Acetylation of asymmetric and symmetric dimethylarginine: an undercharacterized pathway of metabolism of endogenous methylarginines. Nephrol Dial Transplant. 2016;31:57–63. doi: 10.1093/ndt/gfv390 26610597 10.1093/ndt/gfv390

[35] Szuba A, Chachaj A, Wrobel T, Dzietczenia J, Mazur G, Antonowicz-Juchniewicz J, et al Asymmetric dimethylarginine in hematological malignancies: a preliminary study. Leuk Lymphoma. 2008;49:2316–20. doi: 10.1080/10428190802510323 19052979 10.1080/10428190802510323

[36] Doroszko A, Niedzielska E, Jakubowski M, Porwolik J, Turek-Jakubowska A, Szahidewicz-Krupska E, et al Elevated plasma ADMA contributes to development of endothelial dysfunction in children with acute lymphoblastic leukemia. Postepy Hig Med Dosw (Online). 2016;70:562–71. 27333926 10.5604/17322693.1203720

[37] Blankenberg S, Barbaux S, Tiret L. Adhesion molecules and atherosclerosis. Atherosclerosis. 2003;170:191–203. 14612198 10.1016/s0021-9150(03)00097-2

[38] Levey AS, Coresh J, Greene T, Stevens LA, Zhang YL, Hendriksen S, et al Using standardized serum creatinine values in the modification of diet in renal disease study equation for estimating glomerular filtration rate. Ann Intern Med. 2006;145:247–54. 16908915 10.7326/0003-4819-145-4-200608150-00004

[39] Nemeth B, Ajtay Z, Hejjel L, Ferenci T, Abram Z, Muranyi E, et al The issue of plasma asymmetric dimethylarginine reference range—A systematic review and meta-analysis. PLoS One. 2017;12:e0177493 doi: 10.1371/journal.pone.0177493 28494019 10.1371/journal.pone.0177493 PMC5426758

[40] Napora M, Graczykowska A, Prochniewska K, Zdrojewski Z, Calka A, Gorny J, et al Relationship between serum asymmetric dimethylarginine and left ventricular structure and function in patients with endstage renal disease treated with hemodialysis. Pol Arch Med Wewn. 2012;122:226–34. 22538734 10.20452/pamw.1222

[41] Aucella F, Maas R, Vigilante M, Tripepi G, Schwedhelm E, Margaglione M, et al Methylarginines and mortality in patients with end stage renal disease: a prospective cohort study. Atherosclerosis. 2009;207:541–5. doi: 10.1016/j.atherosclerosis.2009.05.011 19501358 10.1016/j.atherosclerosis.2009.05.011

[42] Nijveldt RJ, Teerlink T, Van Der Hoven B, Siroen MP, Kuik DJ, Rauwerda JA, et al Asymmetrical dimethylarginine (ADMA) in critically ill patients: high plasma ADMA concentration is an independent risk factor of ICU mortality. Clin Nutr. 2003;22:23–30. 12553946 10.1054/clnu.2002.0613

[43] Siegerink B, Maas R, Vossen CY, Schwedhelm E, Koenig W, Boger R, et al Asymmetric and symmetric dimethylarginine and risk of secondary cardiovascular disease events and mortality in patients with stable coronary heart disease: the KAROLA follow-up study. Clin Res Cardiol. 2013;102:193–202. doi: 10.1007/s00392-012-0515-4 23073705 10.1007/s00392-012-0515-4

[44] Cavalca V, Veglia F, Squellerio I, De Metrio M, Rubino M, Porro B, et al Circulating levels of dimethylarginines, chronic kidney disease and long-term clinical outcome in non-ST-elevation myocardial infarction. PLoS One. 2012;7:e48499 doi: 10.1371/journal.pone.0048499 23185262 10.1371/journal.pone.0048499 PMC3501498

[45] Bode-Boger SM, Scalera F, Ignarro LJ. The L-arginine paradox: Importance of the L-arginine/asymmetrical dimethylarginine ratio. Pharmacol Ther. 2007;114:295–306. doi: 10.1016/j.pharmthera.2007.03.002 17482266 10.1016/j.pharmthera.2007.03.002

[46] Giles GI. The redox regulation of thiol dependent signaling pathways in cancer. Curr Pharm Des. 2006;12:4427–43. 17168752 10.2174/138161206779010549

[47] Sydow K, Munzel T. ADMA and oxidative stress. Atheroscler Suppl. 2003;4:41–51. 14664902 10.1016/s1567-5688(03)00033-3

[48] Antoniades C, Shirodaria C, Leeson P, Antonopoulos A, Warrick N, Van-Assche T, et al Association of plasma asymmetrical dimethylarginine (ADMA) with elevated vascular superoxide production and endothelial nitric oxide synthase uncoupling: implications for endothelial function in human atherosclerosis. Eur Heart J. 2009;30:1142–50. doi: 10.1093/eurheartj/ehp061 19297385 10.1093/eurheartj/ehp061

