# Supplementary material for: Asymmetric and symmetric dimethylarginines and mortality in patients with hematological malignancies—A prospective study
Source: PLoS One. 2018 May 22;13(5):e0197148. doi: 10.1371/journal.pone.0197148 (PMC5963779; doi:10.1371/journal.pone.0197148)
Supplement: S1 Table — ns- statistically not relevant (DOCX) [file pone.0197148.s001.docx]

|  | **L-arginine** | **CIT** | **sVCAM-1** |  | **L-arginine** | **sVCAM-1** |
| --- | --- | --- | --- | --- | --- | --- |
| **DMA in AML group** | ns | ns | ns | **CIT in AML group** | ns | ns |
| **DMA in nHL group** | ns | ns | r=0.44  p=0.015 | **CIT in nHL group** | r=0.42  p=0.02 | ns |
| **DMA in CLL group** | ns | ns | ns | **CIT in CLL group** | r=0.49  p=0.009 | ns |
| **DMA in control group** | ns | r=0.53  p<0.001 | ns | **CIT in control group** | ns | r= -0.37  p=0.012 |
